# Endoscopic submucosal dissection of esophageal granular cell tumor

**DOI:** 10.1186/1477-7819-12-221

**Published:** 2014-07-17

**Authors:** Wei Lu, Mei-Dong Xu, Ping-Hong Zhou, Yi-Qun Zhang, Wei-Feng Chen, Yun-Shi Zhong, Li-Qing Yao

**Affiliations:** 1 Department of General Surgery, Zhongshan Hospital, Fudan University, 180 FengLin Road, Shanghai 200032, PR China; 2 Endoscopy Center and Endoscopy Research Institute, Zhongshan Hospital, Fudan University, 180 FengLin Road, Shanghai 200032, PR China

**Keywords:** Endoscopic submucosal dissection, Esophagus, Granular cell tumor, Minimally invasive, Abrikossoff tumor

## Abstract

**Background:**

Esophageal granular cell tumor (GCT) is a rare benign tumor with malignant potential. With wide application of endoscopic techniques, the esophageal GCT discovery rate and treatment strategy has changed. This study was to preliminarily evaluate outcomes of endoscopic diagnosis and treatment for esophageal GCT.

**Methods:**

Fourteen patients (eight men, six women; median age, 48.5 years) with esophageal GCT diagnosed and treated by esophageal endoscopy. Esophagoscopy, endoscopic ultrasound (EUS), and endoscopic submucosal dissection (ESD) techniques were employed in diagnosis and resection.

**Results:**

Esophageal GCTs are tumors which arise from the submucosal layer, and vary in color but with a yellowish color on endoscopy being most common. On EUS, features were homogenous (ten cases) or mildly heterogeneous (four cases) hypoechoic solid pattern originating from the muscularis mucosa (six cases) or submucosal layer (eight cases) of the esophageal wall. Tumors ranged from 4 to 26 mm (mean 12.1 mm). ESD was performed in all patients without complication. Clinical diagnosis was confirmed by pathology and immunohistochemical examination (positive for S-100 and vimentin). The *en bloc* resection rate was 92.9% (13/14) pathologically. Operation time was 25 to 60 minutes, mean 38.2 ± 10.1 minutes. No recurrence was observed during a mean follow-up of 16.6 ± 12.7 (range, 4 to 40) months.

**Conclusions:**

Esophagoscopy and EUS increased the esophageal GCT discovery rate, and its features were summarized. Minimally invasive ESD is feasible and safe for excisional biopsy, providing pathological diagnosis and treatment.

## Background

Esophageal granular cell tumor (GCT) is a submucosal tumor (SMT) probably originating from the Schwann cell. It mainly affects the submucosal layer, and less commonly the mucosal layer and muscularis propria [1]. Despite the low morbidity, esophageal GCT is one of the most frequently seen esophageal stromal tumors, second only to leiomyoma [2]. Patients with esophageal GCTs are usually asymptomatic; and the lesions are usually discovered incidentally during esophagogastroduodenoscopy (EGD). Fine-needle aspiration cytology under endoscopic ultrasound (EUS) guidance can be used to obtain tissue from these subepithelial tumors, but the small specimens obtained by needle aspiration are generally too small to make a definite histopathological diagnosis.

It has been suggested that asymptomatic esophageal GCTs smaller than 10 mm could be followed up with periodic endoscopy and/or EUS [3]. Although esophageal GCTs are usually benign lesions, malignant transformation has been reported even in those smaller than 10 mm [4,5]. Moreover, some patients became distressed and desperately sought efficacious resection during follow-up time. The traditional treatment, surgical resection, is invasive and caused significant trauma [6]. Several less invasive endoscopic approaches to remove SMTs are currently available including diathermy loop, endoscopic band ligation, and endoscopic mucosal resection (EMR); [7-9] but they often lead to serious complications, such as incomplete resection or perforation. The new emerging endoscopic technique, endoscopic submucosal dissection (ESD) is now an option for these patients. The aim of our study was to evaluate the endoscopic features for the diagnosis of GCT and the efficacy and safety of ESD techniques for GCT.

## Methods

### Patient identification

We reviewed the Zhongshan Hospital endoscopic therapy database. A total of 14 patients with confirmed histological diagnosis of esophageal GCTs had endoscopic diagnosis and ESD resection at our institute from 2006 to 2011. Patient medical records were reviewed for demographic data including age, gender, and indications for endoscopy. We obtained institutional review board approval for the study. Informed consent for all procedures, including gastroscopy, EUS, and ESD was obtained from each patient.

### Inclusion and exclusion criteria

Patient age from 18 to 75; esophageal submucosal lesion found by endoscopy with size evaluated as less than 30 mm and the patient willing to be treated by ESD technique. Patients with any abnormality in blood clotting, biochemistry or blood cell count were excluded. Patients with esophageal venous varication disease, esophageal stricture disease or history of esophageal surgery were also excluded.

### Endoscopic diagnosis and treatment protocol

Endoscopic and EUS reports and images were reviewed for tumor size, appearance, location, and therapy.

EUS was performed before treatment with a high-frequency miniprobe (UM-2R, 12 MHz; UM-3R, 20 MHz, Olympus Optical Co, Ltd., Tokyo, Japan) to evaluate the tumor origin and size. All available EUS images were reviewed blindly by one proficient endoscopist.

A transparent cap (D-201-11802, Olympus, Olympus Optical Co, Ltd., Tokyo, Japan and for all Olympus products cited below) was attached to the front of the endoscope. Additional equipment and accessories included a high-frequency generator (ICC-200, ERBE, Tübingen, Germany), argon plasma coagulation (APC) unit (APC300, ERBE, Tübingen, Germany), injection needle (NM-4 L-1, Olympus), hook knife (KD-620LR, Olympus), insulated-tip knife (KD-611 L, IT2, Olympus), hot biopsy forceps (FD-410LR, Olympus), and hemostatic clip (HX-600-135, Olympus). An insufflator (Olympus) was used for carbon dioxide gas insufflation during the procedure. A mixed solution of saline with indigo carmine (0.5%) and epinephrine (0.0005%) was prepared for submucosal injection.

Patients were treated under general anesthesia. The resection border was marked using argon plasma coagulation of the normal mucosa approximately 5 mm from the tumor margin. Submucosal injection of the mixed saline solution (5 to 10 ml) was then performed to lift the lesion. After sufficient lifting of the submucosal layer and mucosa, the hook-knife was used to cut open the mucosa in a circumferential incision around the lesion outside the marking dots and then extend into the submucosal layer. The circumferential incision around the lesion was made as deep as the muscularis propria if necessary and dissection was then performed under direct vision to achieve complete *en bloc* resection. The tumor was dissected outside the capsule, and the mixed saline solution was injected repeatedly during the dissection when necessary. Larger vessels or arteries with high bleeding risk were coagulated using hemostatic forceps. The resultant artificial ulcer was managed routinely with APC to prevent delayed bleeding, and hemoclips were used to close the deeply dissected areas when necessary. The time from the marking dots to complete resection of the tumor was recorded.

### Histopathological evaluation

Routine H&E staining and additional immunohistochemistry (IHC) were performed and observed under an optical microscope for evaluation and differential diagnosis. IHC examination included smooth muscle actin, muscle-specific actin, desmin, CD34, S-100, vimentin, CD117, neuron-specific enolase, CD68, and Ki-67. Complete resection was defined as a resected specimen with tumor-free lateral and vertical margins. Incomplete resection was defined as a specimen with histologically positive margins.

### Outcome measurement and follow-up

Esophagogastric endoscopy was performed three and six months after ESD, and then yearly thereafter. Endoscopic forceps biopsies of the operation site were not routinely performed except as necessary when suspicious lesions were observed, and then, additional EUS examination was performed to further diagnose suspected recurrence.

### Statistical analysis

The median of continuous variables was used to present data. The difference in tumor origin on EUS, esophageal location, and time required for resection were compared between the small tumor (≤10 mm in maximal diameter) and large tumor (>10 mm in maximal diameter) groups using a nonparametric two-independent-samples test. A *P*-value less than 0.05 was taken to indicate significant difference. Statistical analysis was performed using the SPSS statistical analysis program (SPSS Inc., Chicago, IL, USA).

## Results

**Figure 1:**
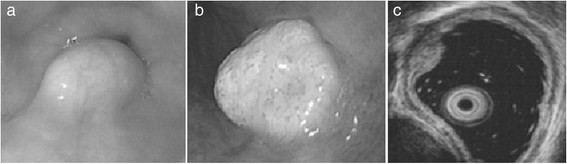
**Endoscopic and endoscopic ultrasound (EUS) image of different esophageal granular cell tumors (GCTs). (a)** Small yellowish peanut-like half protrusion under the smooth overlying mucosa. **(b)** A submucosal knurl with a small tuberculum. **(c)** EUS view of esophageal GCT (mildly heterogeneous solid pattern originating from the muscularis mucosa layer).

A total of 14 patients aged 29 to 75 years with GCTs of the esophagus were identified including 8 men and 6 women. The patient clinical data are listed in Table 1 and Table 2. The median age at time of diagnosis was 48.5 years. Five patients suffered epigastric discomfort; three had retrosternal discomfort and regurgitation; two suffered regurgitation and belching; two suffered from dysphagia, and the remaining two non-symptomatic patients were discovered by routine physical examination and gastric endoscopy. All patients had a single esophageal GCT. Eight were in the distal thoracic or abdominal part of the esophagus, three in the proximal part, and three in the mid-thoracic esophagus.The endoscopic appearance of esophageal GCTs showed variation in size, location, and coating layer of mucosa in our study. All GCTs were described as well circumscribed raised lesions under the mucosal layer. Eight cases were small tumors, most of which (five cases) exhibited a small yellowish peanut-like half protrusion under the smooth overlying mucosa (Figure 1a). However, the larger tumors (>10 mm) appeared as a submucosal knurl with a small tuberculum (one case, Figure 1b) or smooth edge (five cases). Among them, four cases had a yellowish or pale color. Among all GCTs, three cases were covered with a rough mucosal layer due to mucosal inflammatory disease of the esophagus and were of a similar color to the surrounding mucosa. One case showed the same color as the mucosa without inflammatory changes.

**Table 1:** Patient clinical data

**Patient**	**Age**	**Sex**	**Symptom**	**Esophagus site (cm)**	**Size (mm)**	**EUS depth**^**a**^	**EUS echo**^**b**^
1	47	Male	Retrosternal discomfort, regurgitation	30	5	mm	Homogenous
2	35	Female	Dysphagia	23	10	sm	Heterogeneous
3	45	Male	Epigastric discomfort	40	6	sm	Homogenous
4	53	Male	Retrosternal discomfort, regurgitation	38	7	mm	Homogenous
5	75	Female	Regurgitation, belching	37	6	mm	Heterogeneous
6	29	Male	Epigastric discomfort	40	15	sm	Homogenous
7	55	Female	None	19	8	mm	Homogenous
8	50	Female	Regurgitation, belching	35	4	mm	Homogenous
9	59	Female	Epigastric discomfort	20	23	sm	Heterogeneous
10	32	Female	None	30	6	sm	Homogenous
11	51	Male	Retrosternal discomfort, regurgitation	35	18	sm	Homogenous
12	58	Male	Epigastric discomfort	37	26	sm	Heterogeneous
13	48	Male	Dysphagia	25	15	mm	Homogenous
14	42	Male	Epigastric discomfort	35	20	sm	Homogenous

^a^mm, tumor originated from the muscularis mucosa layer; sm, tumor originated from the submucosal layer.

^b^All were hypoechoic, four were mildly heterogeneous, eight were homogeneous.

EUS, endoscopic ultrasound.

**Table 2:** Patient clinical data

**Patient**	**Operation time (minutes)**	**Pathological depth**	**Pathological cutting edge**	**Follow-up (months)**
1	25	No data	Negative	40
2	30	sm	Negative	36
3	40	sm	Negative	30
4	30	No data	Negative	30
5	35	mm	Negative	18
6	45	sm	Negative	18
7	40	mm	Negative	18
8	30	mm	Negative	12
9	45	sm	Positive	6
10	25	sm	Negative	6
11	35	sm	Negative	6
12	60	No data	Unrecognized eschar	5
13	45	No data	Negative	4
14	50	sm	Negative	4

mm, muscularis mucosa; sm, submucosa.

EUS was performed in all patients who were diagnosed with SMT in our institute. On EUS, esophageal GCT in our study appeared as a homogenous (ten cases) or mildly heterogeneous (four cases) hypoechoic solid pattern originating from the muscularis mucosa (six cases) or submucosal layer (eight cases) of the esophageal wall, with smooth edges despite the small tubercular aspect on endoscopy (Figure 1c). The maximum diameter of these lesions ranged from 4 to 26 mm with a median size of 12.1 mm. The tumors from the submucosal layer (8 cases, mean 15.5 ± 7.6 mm) were significantly smaller than those from the muscularis mucosa layer (6 cases, mean 34.2 ± 7.4 mm), but there was no obvious difference in size according to the location of the lesions (Table 3).

**Table 3:** Comparison of tumor size according to the location and origin of the tumor

	**Classification**	**Number**	**Size**^**a **^**(mean ± deviation) mm**	***P*-value**
Location in the esophagus	Distal part (>32 cm)	8	12.8 ± 8.1	0.699
	Proximal part (≤32 cm)	6	11.2 ± 6.8	
Depth of the tumor origin	Submucosal layer	8	15.5 ± 7.6	0.027
	Muscularis mucosa layer	6	34.2 ± 7.4	
Total		14	12.1 ± 7.3	

^a^Size detected by the endoscopic ultrasound (EUS).

Endoscopic forceps biopsy was not routinely performed in our institute when the lesion was recognized as a submucosal mass, because of the high risk of destroying the tumor integrity and causing bleeding. However, three tumors mimicked mucosal lesions because of their rough overlying mucosa, and thus endoscopic forceps biopsies were performed. Histological diagnosis of GCT was confirmed via biopsy in only one of these.

**Figure 2:**
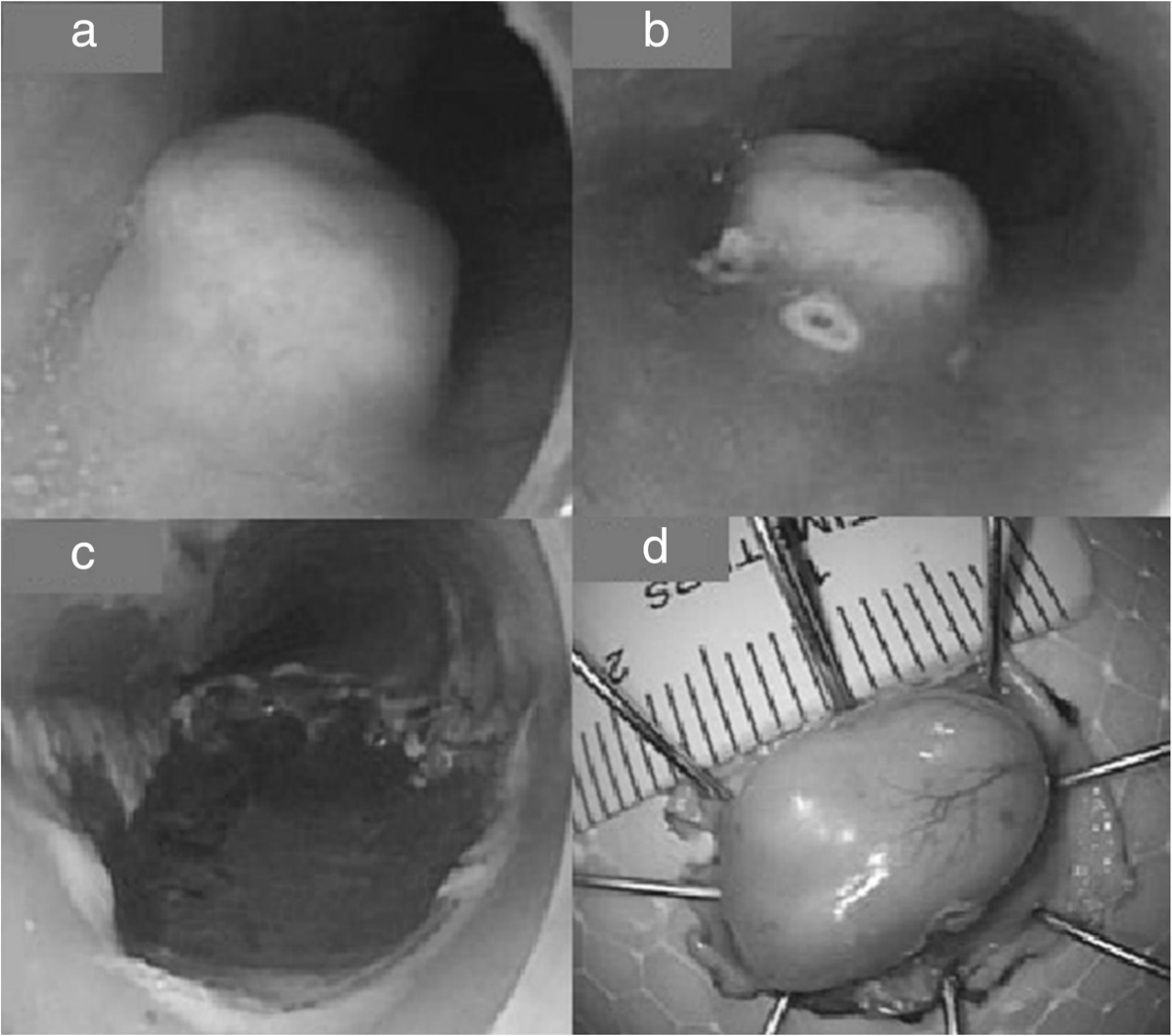
**Endoscopic submucosal dissection (ESD) procedure for esophageal granular cell tumor (GCT). (a)** Submucosal injection of mixed saline solution. **(b)** Circle labels the edge of the GCT. **(c)** The surface of the wound (muscularis propria layer). **(d)** The specimen fixed and measured.

**Figure 3:**
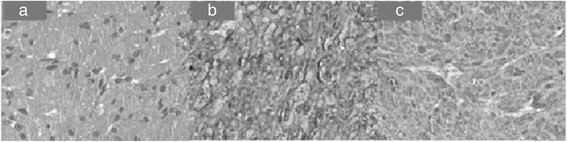
**Histopathological evaluation of the esophageal granular cell tumor (GCT) (×100). (a)** The H&E stain of esophageal GCT. **(b)** Immunohistochemistry (IHC) positive for S-100. **(c)** IHC positive for vimentin.

Standardized ESD procedures were performed in all patients in our study. The wound surface of the esophageal GCT during ESD procedure is shown in Figure 2a to c and the excised specimen is showed in Figure 2d. The procedure time was from 25 to 60 minutes (mean 38.2 ± 10.1 minutes). As shown in Table 4, it took significantly more time (*P* < 0.05) to remove the large tumors (6 cases, mean 46.7 ± 8.2 minutes) than the small tumors (8 cases, mean 31.9 ± 5.9 minutes). The procedure time for the tumors in the submucosal layer (8 cases, mean 41.3 ± 11.3 minutes) was more than for tumors in the muscularis mucosa layer (6 cases, mean 34.2 ± 7.4 minutes), but this was not statistically significant. Severe complications of ESD including perforation and delayed bleeding were not observed in all patients. The length of hospital stay was two to four days for each patient.Pathological diagnosis was confirmed in all cases after ESD (Figure 3a). All the lesions were well-circumscribed but without an obvious capsule. The margins were negative in 13/14 cases (complete resection rate 92.9%). One case with an unrecognized eschar in the vertical margins was designated treatment Rx (Resection margin unclear) margin. The pathological diagnosis was supported by IHC with the characteristic positive for S-100 and vimentin (positive in all esophageal GCTs in our study, Figure 3b and c).

**Table 4:** Comparison of the mean operation time by tumor size, location, and depth

	**Classification**	**Number**	**Time cost (mean ± deviation) minutes**	***P*-value**
Size	Small tumor (≤10 mm)	8	31.9 ± 5.9	0.005
	Large tumor (>10 mm)	6	46.7 ± 8.2	
Location in the esophagus	Distal part (>32 cm)	8	40.6 ± 10.5	0.316
	Proximal part (≤32 cm)	6	35.0 ± 9.5	
Depth of the tumor origin	Submucosal layer	8	41.3 ± 11.3	0.181
	Muscularis mucosa layer	6	34.2 ± 7.4	
Total		14	38.2 ± 10.1	

The total follow-up time varied from 4 to 40 (mean 16.6 ± 12.7) months. No recurrent disease was observed during follow-up, including the Rx margin case, within 18 months. A small protuberance of the mucosal scar was found at the operation site in two cases (negative margin) on follow-up examination. Endoscopic forceps biopsy and EUS examination were performed. EUS did not find any deep lesions below the mucosa and the pathologic diagnosis from the forceps biopsy proved that these were inflammatory granulomas.

## Discussion

Esophageal GCT is a type of SMT which is thought to arise from Schwann cells of the submucosal neuronal plexus and is usually a benign neoplasm but has some malignant potential. Approximately 2 to 4% of esophageal GCTs reported in the literature were malignant [10], of which approximately 15% were less than 10 mm in diameter. SMTs may more often be discovered in the esophagus today with the advent of screening gastroscopy. Esophageal GCTs do not exhibit specific features on endoscopy and EUS, and it is difficult to distinguish GCTs from other SMTs, such as leiomyoma or gastrointestinal stromal tumors. Thus histopathological evaluation is necessary for differential diagnosis.

EUS-guided fine needle biopsy or multiple forceps biopsy were employed in an attempt to obtain tissue diagnosis, but the results were not reliable because the tumors were small, underlying the mucosa layer, and biopsy tissue was not adequate. Moreover, there is sampling error when the biopsied tissue does not encompass the areas of malignant change [11]. Complete resection of the lesion is necessary for its accurate characterization and patient treatment.

Open surgery or thoracoscopy is highly invasive and can lead to a decrease in patient quality of life compared to simple endoscopic resection. However, finding these lesions operatively was difficult because they were less than 20 mm in diameter. Endoscopic resection methods can prevent skin scars caused by incisions, reduce the inflammatory response and perceived pain associated with surgical trauma, and lower the risk of postoperative infection. Endoscopic methods are extremely minimally invasive, and are becoming the preferred treatment of benign esophageal tumors including leiomyoma, GCT, and gastrointestinal stromal tumor (GIST).

Currently, traditional polypectomy and EMR are most commonly employed for GCTs. However, complete histological resection may not always be easy to achieve using EMR because most esophageal GCTs are not confined to the mucosa, but rather, involve the submucosa, which results in frequent involvement of the resection margin.

To date, there are several reports showing successful removal of esophageal GCT by EMR, but most of these are small series or single case reports. The maximum number of cases of successful removal of esophageal GCT was ten from twenty-three patients reported from Mayo Clinic. However, EMR was not perfect for endoscopic resection of these lesions, especially for lesions exceeding 10 mm in diameter. There is a high risk of incomplete removal. An attempt to remove a 13-mm esophageal GCT by EMR was unsuccessful in a report from Mayo Clinic [12].

ESD is a method of endoscopic resection that involves circumferential cutting of the mucosa surrounding the tumor followed by dissection of the submucosa beneath the lesion. ESD has the advantage of a high probability of *en bloc* excision and histologically complete resection even in large lesions because the technique involves dissection of the submucosal tissue beneath the lesion [13]. It has been widely accepted for the treatment of early gastric cancer and also used in resection of submucosal tumors. ESD of GCT can provide a definite tissue diagnosis, and a high likelihood of complete removal of the lesion. In contrast to conventional EMR, ESD was able to increase the rates of *en bloc* and histologically complete resection, which may reduce local recurrent rate. In our study, the pathological *en bloc* resection rate was 92.9% (13/14). Only one was not recognizable because the cut edge had an eschar.

We demonstrated that ESD is a safe, effective, and minimally invasive procedure for treating esophageal GCT. Our experience with ESD in 14 esophageal GCTs validates the safety and efficacy in lesions less than 26 mm in size. Given the apparently low risk of this method, it is reasonable to consider ESD the treatment of choice for esophageal GCT.

Bleeding and perforation are the two main complications of ESD of esophageal lesions. Other complications including pneumothorax and subcutaneous emphysema are secondary to perforation. In this study, none of the above complications were observed. Our measures in reducing the complications of ESD were: a) keep the operation field clear and perform all the actions under direct vision, b) prophylactic hemostasis of visualized vessels, c) well selected length and angle of the knife during the operation, and d) after the lesion was removed, hot biopsy forceps coagulation or APC were performed on the whole wound surface. This management strategy can reduce delayed hemorrhage and obliterate the residual lesion if it exists.

The ESD procedure time in this study varied from 25 to 60 minutes, and there was a significant difference in operation time according to tumor size. It took more time to dissect the large tumors and tumors derived from the submucosal layer. The length of hospital stay was two to four days for post-operative observation. The ESD procedure caused little trauma and was relatively inexpensive.

Follow-up ranged from 4 to 40 months (mean duration 16.6 ± 12.7), during which time local recurrence was not observed in any patient at gastroscopy. However, because of the limited number of patients and relatively short follow-up duration, we realize that much longer follow-up may be needed to evaluate long-term results of ESD of esophageal GCT.

## Conclusions

In summary, this study showed that esophageal GCTs did not appear uniform under endoscopy. EUS is an accurate imaging test for detecting the component of the esophageal wall from which the mass arises. Resection by ESD safely and entirely removed tumors without complications and should be the preferred procedure; this also helps to obtain an exact pathological diagnosis. Further investigation is required to assess the long-term outcomes of this method.

## Abbreviations

APC: Argon plasma coagulation; EGD: Esophagogastroduodenoscopy; EMR: Endoscopic mucosal resection; ESD: Endoscopic submucosal dissection; EUS: Endoscopic ultrasound; GCT: Granular cell tumor; GIST: Gastrointestinal stromal tumor; H&E: Hematoxylin and eosin; IHC: Immunohistochemistry; SMT: Submucosal tumor.

## Competing interests

The authors declare that they have no competing interests.

## Authors’ contributions

Conception and design: M-DX, L-QY. Provision of study materials or patients: WL, M-DX, P-HZ, L-QY, Y-QZ, Y-SZ, W-FC. Collection and assembly of data: WL, M-DX. Data analysis and interpretation: WL, M-DX. Manuscript writing: WL, M-DX. All authors read and approved the final manuscript.
